# Investigating the effect of multimedia education based on the health belief model in preventing COVID-19 in pregnant women

**DOI:** 10.1186/s12889-022-14965-1

**Published:** 2023-04-12

**Authors:** Ghodratollah Shakerinejad, Tayebeh Navak, Nasser Hatemzadeh, Mehdi Haghi, Mohammad Hossein Haghigizadeh

**Affiliations:** 1 Health Education Research Department, ACECR-Khuzestan, Ahvaz, Iran; 2 Department of Health Education and Health Promotion, School of Public Health, Ahvaz Jundishapur University of Medical Sciences, Ahvaz, Iran; 3 Department of Health Education and Health Promotion, Social Determinants of Health Research Center, School of Public Health, Ahvaz Jundishapur University of Medical Sciences, Ahvaz, Iran; 4 Department of Public Health, Faculty of Health and Nutrition, Lorestan University of Medical Sciences, Khorramabad, Iran; 5 Department biostatistics, School of health, Ahvaz Jundishapur University of Medical Sciences, Ahvaz, Iran

**Keywords:** COVID-19, Pregnant women, Health belief model, Multimedia, Intervention

## Abstract

**Background:**

Pregnant women are considered one of the high-risk groups during the COVID-19 pandemic, so paying attention to preventive behaviors among them is highly important. This study aimed to examine the effect of multimedia education based on the Health Belief Model (HBM) in preventing COVID-19 among pregnant women.

**Methods:**

This quasi-experimental intervention study was conducted on 120 pregnant women referring to Comprehensive Health Services Centers affiliated with East and West health centres of Ahvaz city, Iran, in 2021. Participants were divided into two control (*n* = 60) and intervention (*n* = 60) groups. A researcher-made questionnaire was used for data collection. The intervention group was given the required educational content using social networks virtually and multimedia in 12 sessions. Both groups were reinvestigated after two months. Data were analyzed using SPSS software version 24, independent t-test and paired t-test tests.

**Results:**

The mean age and mean gestational age of participants were estimated at 28 years old and 18 weeks, respectively. Before the educational intervention, there was no significant difference in mean constructs of HBM. In contrast, the mean of all constructs increased significantly in the intervention group after intervention. The greatest change was related to the constructs of self-efficacy and perceived susceptibility, and the lowest change was related to the perceived barriers construct.

**Conclusion:**

Our findings suggest multimedia education using the HBM to COVID-19 preventive behaviors among pregnant women can benefit behavior change.

**Supplementary Information:**

The online version contains supplementary material available at 10.1186/s12889-022-14965-1.

## Introduction

On December 31, 2019, the first cases of COVID-19 were reported in Wuhan, China. The disease was then named the 2019 novel coronavirus (2019-nCov) [1]. With the rapid spread of the disease in China and then all over the world, the virus was declared a global public health emergency by the World Health Organization (WHO) [2]. Coronavirus disease 2019 (COVID-19) is a viral disease caused by severe acute respiratory syndrome coronavirus 2 (SARS-CoV-2).

During the epidemic of infectious diseases, pregnant women and their fetuses are among the high-risk groups [3]. Due to physiological changes in the immune and cardiopulmonary system during pregnancy, pregnant women are more exposed to severe diseases after being exposed to viruses, especially respiratory viruses, than other women [4]. Hence, there are concerns about the serious consequences of the novel Coronavirus disease 2019 for pregnant women.

Evidence demonstrated that the mortality rate among pregnant women and fetal complications increased during Influenza, Severe Acute Respiratory Syndrome (SARS) and Middle East Respiratory Syndrome (MERS) [1, 5]. Studies in Iran reported that the most common clinical symptoms among pregnant women were fever, cough, muscle pain and sore throat [6, 7], and the most common maternal outcomes were intrauterine distress, PROM, and preterm delivery [8] Nikpour et al. (2021) showed that common manifestations of COVID-19 in pregnant women were fever, cough, and muscle pain, and the most common laboratory results were a decline in blood lymphocytes and an increase in blood CRP [6]. A systematic review showed that the most common clinical symptoms were fever, cough, and sore throat, and the most common maternal outcomes were intrauterine distress, PROM, and preterm delivery. The most common complications of preterm infants included fetal distress, low birth weight, and bacterial pneumonia [7]. Therefore, pregnant women and newborn babies should be prioritized when adopting measures/ interventions to prevent and manage COVID-19 [9].

Preventive measures to reduce the probability of infection include getting vaccinated, staying at home, wearing a facemask in public places, avoiding going to crowded places, sticking to physical distancing, ventilating indoor spaces, washing hands regularly with soap and water, sticking to respiratory hygiene and avoiding of shaking hands, touching the eyes, nose or mouth with unwashed hands [10]. Because there is no definitive treatment or vaccine for COVID-19, how people behave is of the utmost importance to controlling and preventing the disease [11]. Health education using various methods is a suitable means to motivate and amend inappropriate performance because health education is nothing but the science and art of making people pay attention to a learning process to create desirable behavior in order to achieve health [12].

Today, researchers in the field of health education have developed effective models in order to achieve the goal of changing behavior using different theories of psychology and social sciences [13]. The HBM is one of the most effective and widespread psychosocial approaches to describing health-related behaviors [14]. According to this model, a person’s behaviour is based on her/his knowledge and beliefs. The model also emphasizes that a person’s perception of vulnerability towards a problem will affect her/his decision regarding health behaviors [15].

The development of information technology has increased the flexibility of the learner and the teacher and provided feedback in the virtual space, which in turn has led to the expansion of the use of modern educational methods [16]. Multimedia in the computer sense means the use of audio and video modalities (fixed and moving) to convey a message with a specific purpose, as well as the use of multimedia tools such as audio and video effects, infographics, short videos, etc., in order to convey the desired concepts [17]. Moreover, Multimedia is a structure that communicates with the audience by combining visual and audio graphic elements and various types of videos along with written elements. The audience engages with these concepts in different ways, creating a kind of two-way interaction between the creator and the audience [18]. Multimedia combines text, graphics, sound, animation and video images provided to the user through a computer or other electronic equipment. In fact, the information delivery through multimedia for message delivery, or the simultaneous influence of two or more media on a person to achieve a specific goal, is called multimedia. An educational multimedia message is communication using words and pictures, resulting in active learning [19]. One of the advantages of multimedia learning is the possibility of teaching from any place and at any time. It allows individuals to obtain the information they require easily. It also reduces or eliminates many educational barriers. Its other advantages include speed, the scope of publication, easy publication, variety and volume of content, quick access and coverage of a broad range of audiences. This type of educational method is comprehensive and based on people’s individual needs, interests and abilities. It also stimulates learners’ motivation for active participation and leads to self-directing learning [20]. The learner can pursue her/his studies despite work and family busyness or difficult conditions and have enough time to interpret, understand and respond [21]. The most important reason for using educational software, podcasts, and multimedia is to use multimedia facilities, including images, sound, text, etc., and simultaneously to teach individuals, to create a fully interactive and user-friendly educational environment and the possibility of repeating educational materials, reducing costs of reproduction and publication and education, the possibility of using multiple and scattered contacts regardless of time and place limitations [22].

Educational software, clips and educational motions, audio files and podcasts, and files presenting educational clips are examples of multimedia. Different studies have demonstrated the effectiveness of using multimedia in educational interventions [23–25].

Pregnant women are a vulnerable group in society that needs more care and education to prevent diseases, especially Covid-19. Therefore, the training should be designed so that these individuals can achieve it with no cost/low cost and in less time. This study aimed to examine the effect of multimedia education based on the Health Belief Model (HBM) in preventing COVID-19 among pregnant women referring to healthcare centers in Ahvaz, Iran, in 2021.

## Methods

### Study design and setting

We conducted a quasi-experimental intervention study among 120 pregnant women referring to Comprehensive Health Services Centers affiliated with East and West health centers of Ahvaz city, Iran, in 2021. Participants were divided into two control (*n* = 60) and intervention (*n* = 60) groups.

The sample size calculation was based on a similar study [26], and with 95% confidence, 90% statistical power and 10% error, the sample size was calculated as 54 participants (54 in each group). With an expected drop-out rate of 10%, the sample size for each group was therefore determined at 60. Sampling from the two health centers of East and West was conducted using simple random sampling. West Health Center was considered as an intervention, and East Health Center was considered as a control group. From comprehensive health services centers, two health centers based on the random cluster sampling method and 8 centers (4 centers in the east and 4 centers in the west) based on the coverage of the sample size were selected.

In this study, inclusion criteria were gestational age up to 28 weeks, familiarity and ability to download and install smartphone apps, no history of COVID-19 infection, and not having underlying diseases related to pregnancy (e.g. hypertension, hyperthyroidism, etc.). Exclusion criteria were unwillingness to participate in the study, incomplete information in the questionnaire, migration, absence in educational sessions (more than 2 sessions), or contracting COVID-19. The educational content includes educating high-risk groups about Covid-19, maternal and neonatal outcomes associated with Covid-19, prevention methods such as the correct way to wear a face mask, wash hands properly, accommodate social distancing, and how to receive services and electronic consultations during quarantine, etc. The educational intervention was performed through the creation of a WhatsApp group for six weeks during 12 sessions (each session takes about one hour) using multimedia and in the form of group discussions, broadcasting short films from midwifery and gynecologist experts, motion graphics, audio and video clips, short text, educational slides, educational message in the form of a poster based on the instructions of the Ministry of Health.

### Data collection tools

This study’s data collection tools consisted of three sections. The first section was related to socio-demographic questions (e.g. age, education status, employment status, gestational age, vaccination history and reason for not getting vaccinated).

The second section was related to constructs of HBM consisting of perceived susceptibility (There is a possibility that I will get infected with Covid-19 during pregnancy) (5 items with scores ranging from 5 to 20), perceived severity (If I infected with Covid-19, I might have more severe symptoms) (5 items with scores ranging from 5 to 20), perceived barriers (I think that compliance with the health recommendations against Covid-19 is costly) (5 items with scores ranging from 5 to 20), perceived benefits (The cost of preventing Covid-19 is lower than its cost of treatment) (5 items with scores ranging from 5 to 20), self-efficacy (Although hand washing is a time-consuming process for me, I can do it.) (7 items with scores ranging from 7 to 35), cues to action (To get information on ways of preventing the spread of Covid-19, I take help from the recommendations of physicians and health experts) (6 items with scores ranging from 6 to 30). The items were rated on a 5-point Likert Scale with scores ranging from 1 (strongly agree) to 5 (strongly disagree).

The third section was a preventive behavior questionnaire (I use a disposable face mask when leaving the house). This questionnaire consisted of 15 questions with scores ranging from 0 to 60. The items The items were rated on a 5-point Likert Scale with scores ranging from 0 (always) to 4 (never). The scoring was reversed for perceived barriers.

In this study, the content reliability of the questionnaires was confirmed by a panel 14 experts consisting of specialists and experts in the field of health education and health promotion (*n* = 11), infectious disease specialist (*n* = 1) and gynecologists (*n* = 2). A pilot study was conducted among 30 pregnant women in order to check the clarity and readability of the questionnaire. The Cronbach’s alpha of the HBM constructs was 0.7 for perceived susceptibility, 0.8 for perceived severity, 0.7 for perceived benefits, 0.74 for perceived barriers, 0.7 for cues to action, 0.81 for self-efficacy and 0.84 for perceived behavior, indicating good internal consistency reliability.

Due to the COVID-19 pandemic, the questionnaires were made available to the participants through Google Forms and virtual networks (e.g. WhatsApp). The participants were asked to complete the questionnaire through the messenger network) whats app). Educational content was designed based on the pre-test analysis and was performed virtually by creating a WhatsApp group for 6 weeks. The educational content was conducted using multimedia within 12 sessions of one hour and in the form of group discussions and video calls with the members. In this study, multimedia was provided as a combination of short films from midwives and gynecologists, motion graphics, audio and video clips, short text, educational slides, and educational messages in the form of posters. All of which were presented based on the instructions sent by the Ministry of Health’s Avasalamat website. In order to involve the participants and their regular attendance in the virtual educational sessions, a time was assigned for the potential questions and answers at the end of each session. Two months after the end of the educational course, the questionnaire was given again to intervention and control groups.

### Data analysis

Data were analyzed using SPSS software version 24. Descriptive statistics such as mean ± Standard Deviation (SD), frequency and percentage for (qualitative variables) were used to describe the quantitative and qualitative variables. Paired t-Test was used to compare quantitative variables in each group. An Independent t-test was applied to compare the mean between the two groups, and a Chi-square test was used to compare the frequency of variables between the two groups. All tests were performed at a level of confidence of 95%.

## Results

The socio-demographic characteristic of participants is presented in Table 1. A total of 120 pregnant women were included in this study. The mean ± SD age of pregnant women in the intervention and control groups was estimated at 27.55 ± 6.55 and 27.83 ± 6.51, respectively, and this difference was not statistically significant (*P* = 0.62). Gestational age in the intervention and control groups was 17.56 ± 5.95 and 18 ± 1, respectively. Approximately 60% of participants had a high school diploma or less and were housewives (75%). In the intervention group, 53.3% had a vaccination history, while in the control group, 63.3% had a vaccination history. 53.6% in the intervention group and 54.5% in the control group reported refusing to get vaccinated due to its complications.

**Table 1:** Distribution of absolute and relative frequency of qualitative demographic variables in control and intervention group

Variable	Modes	Intervention group	Control group	*p-****value***
		Frequency (%)	Frequency (%)	
**Education status**	**High school diploma or less**	35 (58.3)	44 (73.3)	0.118
	**Associate’s degree or bachelor’s degree**	23 (38.3)	16 (26.7)	
	**Master’s or PhD degree**	2 (3.3)	0 (0)	
**Employment status**	**Housewife**	45 (75)	52 (86.7)	0.346
	**Employee**	10 (16.7)	5 (8.3)	
	**Self-employed**	4 (6.7)	3 (5)	
	**Other**	1 (1.7)	0 (0)	
**Vaccination history**	**Yes**	32 (53.3)	38 (63.3)	0.267
	**No**	28 (46.7)	22 (36.7)	
**Reason for not getting vaccinated**	**Fear of complications**	15 (53.6)	12 (54.5)	0.926
	**Crowded health centers**	2 (7.1)	1 (4.5)	
	**Lack of knowledge**	1 (3.6)	1 (4.5)	
	**Family and friends’ opposition**	1 (3.6)	2 (9.1)	
	**Other**	9 (32.1)	6 (27.3)	

As shown in Table 2, before the educational intervention, there was no significant difference in the construct mean of HBM between the control and intervention groups (*P* > 0.05). In contrast, after educational intervention, a significant difference was observed between the control and intervention groups (*P* < 0.05), according to the independent t-test.

**Table 2:** Average constructs of the health belief model before and after the educational intervention in two groups

Variable	Group	Before intervention	After intervention	Intragroup comparison^**^
		Mean ± SD	Mean ± SD	
**Perceived susceptibility**	**Intervention**	22.15 **±** 2.64	22.45 **±** 1.22	.000
	**Control**	22.48 **±** 2.28	22.38 **±** 2.09	.433
	Intergroup comparison^*^	.461	.000	
**Perceived severity**	**Intervention**	19.63 ± 3.47	23.55 ± 2.28	.000
	**Control**	20.41 ± 4.31	20.58 ± 4.15	.321
	**Intergroup comparison**	.278	.000	
**Perceived barriers**	**Intervention**	15.8 ± 3.73	23.38 ± 2.08	.000
	**Control**	17.05 ± 3.95	17.3 ± 3.77	.255
	**Intergroup comparison**	.078	.000	
**Perceived benefits**	**Intervention**	21.56 ± 2.77	24.41 ± .88	.000
	**Control**	22.41 ± 2.94	22.51 ± 2.79	.226
	**Intergroup comparison**	.079	.000	
**Self Efficacy**	**Intervention**	29.83 ± 3.6	34.25 ± 1.55	.000
	**Control**	30.3 ± 4.1	30.4 ± 3.86	.277
	**Intergroup comparison**	.511	.000	
**Cue to action**	**Intervention**	25.38 ± 2.48	29.25 ± 1.06	.000
	**Control**	26.21 ± 2.78	26.41 ± 2.77	.153
	**Intergroup comparison**	.086	.000	
**Behavior**	**Intervention**	51.05 ± 7.01	57.35 ± 3.33	.000
	**Control**	48.53 ± 10.78	48.95 ± 10.17	.678
	**Intergroup comparison**	.137	.000	

^**^Independent t-test

^*^Paired sample t-test

## Discussion

This study was designed to examine the effect of multimedia education based on the Health Belief Model (HBM) in preventing COVID-19 among pregnant women in Ahvaz, Iran, in 2021. Our study showed that before the intervention, none of the HBM constructs was significant in the intervention and control groups, and both groups had the same status in terms of underlying variables.

In this study, the mean score of perceived susceptibility in the intervention group increased from 22.15 before the intervention to 24.45 after the intervention. This can be due to the possibility of negative consequences or representing complications and dangers caused by COVID-19 for pregnant mothers and their fetuses on social media. The higher the perceived sensitivity, the higher the probability of adopting preventive behaviors because perceived susceptibility is considered one of the effective factors in adopting preventive behaviors [27]. While our findings were not in line with previous studies in Iran [28, 29], they were consistent with several studies that demonstrated increased perceived susceptibility in pregnant women after intervention [26, 30–33]. Considering that high perceived susceptibility is necessary to promote people’s motivation to adopt preventive health behavior, part of the educational program regarding issues such as the prevention of Covid-19 should be focused on this construct.

In the present study, the mean score of perceived severity increased significantly from 19.6 before intervention to 23.55 after intervention in the intervention group, which is consistent with previous studies [26, 34–37]. In contrast, our results were not in agreement with some studies [28, 38]. The similar clinical symptoms and features of Covid-19 to flu and colds may reduce the perceived severity, so increasing awareness through media and multimedia can increase people’s awareness of the severity of Covid-19. In the present study, the complications of COVID-19 for pregnant women and the risk of unknown complications for the fetus were important factors in improving the perceived severity of pregnant women. Moreover, since covid-19 symptoms in pregnant women are more severe, they may consider contracting COVID-19 to themselves and their fetuses deadly and dangerous. Hence, perceived severity can lead to adopting COVID-19 preventive behavior.

Regarding perceived benefits, the mean score of this construct increased in the intervention group after the intervention. For more effectiveness of the perceived benefits construct, health educators should focus on specifying the precise behavior that needs to be done and highlighting its benefits [39]. Our result is in tune with previous studies [26, 35, 40]. It seems that the awareness and information about the disease, in part, shift the belief and attitudes of the studied society towards the benefits of Covid-19 preventive behaviours. Another reason may be the extensive information through the national and social media and the guideline of the World Health Organization that the only way to overcome this disease is compliance with personal hygiene and hand washing. Having perceptions about the effects of regular hand washing and the use of Personal Protective Equipment (PPE) such as face masks and disposable gloves can lead to high perceived benefits and, as a result, a strong motivation to take preventive measures against Covid-19. In contrast, our result was not found in other studies [34, 41]. This may be due to differences in the studies’ methodology and participants’ sociodemographic characteristics, as well as the lack of use of multimedia in other studies.

In the current study, the mean score of perceived barriers increased in the intervention group after intervention. Perceived barriers refer to a person’s beliefs about the actual costs and obstacles to performing a recommended health action. A person weighs the effectiveness of the actions against the perception that they may be expensive, dangerous, unpleasant, time-consuming, or inconvenient [39]. Also, the perceived barriers are the factors preventing the adoption of disease prevention behaviors, which makes the pregnant woman perform an analysis of the benefits of the preventive behavior for the costs, risks, fetal complications, fatigue, and time, and based on that, adopts a health behavior. This finding is in agreement with studies conducted on different populations [26, 34, 42]. However, this is not in line with the study of Rafiei et al. [41]. There is an inverse association between perceived barriers and fatalistic beliefs about Covid-19. Perceived barriers are important and effective health belief model constructs because people must overcome behavioral barriers despite their intrinsic desire to engage in preventive behaviors. Some barriers during the Covid-19 are environmental obstacles such as the lack of face masks, alcohol pads and disinfectants. A shortage of face masks has been observed in most countries [43, 44]. It seems that health educators must reduce such barriers so that these barriers do not prevent a person from performing the recommended actions/behaviors, and do this by reassurance, resolving misunderstandings, or providing incentives.

We found that the mean score of self-efficacy increased from 29.83 before intervention to 32.25 after intervention in the intervention group. A similar result was found in studies conducted in different parts of Iran and with different populations [26, 45]. However, this is not consistent with the study of Hashemi et al. and Nik Sadat et al. [26, 45]. This difference can be due to differences in the study population and educational programs. It seems that those with more self-efficacy would have more resistance and persistence to cope with challenges. Therefore, improving this construct can make problems and challenges easier for them. Moreover, considering the role of this construct in empowering pregnant women to adopt COVID-19 preventive behaviors, more attention should be paid to designing educational programs. Since the improvement of self-efficacy appears to influence the promotion of other constructs, greater attention should be given to it when conducting the intervention.

We found that the mean score of cues to action increased in the intervention group after intervention. Cue to action refers to the stimulus needed that makes a person feel the need to perform an action. Such cues can be internal (e.g., understanding a physical condition) or external (e.g., interpersonal interactions, media communication, or receiving a postcard from a doctor to follow up on a test) [39]. This is congruent with previous studies in which similar results were found [26, 27, 34].

Promoting the adoption of preventive behaviors through mass media, emphasizing the sense of social responsibility and sending notifications through mobile phones and social media can improve COVID-19 preventive behaviors [46]. Using preventive behaviors by doctors and health experts and promoting and advertising preventive behaviors on social networks, mass media, and sending notifications via mobile phones, as well as internal factors such as fear of infection and quarantine, can increase preventive behaviors of COVID-19. After the educational intervention, COVID-19 preventive behaviours in the intervention group increased significantly, indicating the effectiveness of the educational intervention. Similar results were also found in previous studies [33, 37, 47, 48]. In the present study, the preventive behaviors were wearing a disposable face mask when leaving the house, perfectly wearing a face mask, washing hands correctly, using social networks to greet and communicate with friends and family members, compliance with social distancing when leaving the house, Counseling via internet and/ or telephone and shopping online.

## Conclusion

According to our results, the intervention group adhered to preventive programs and the mean score of constructs increased significantly in this group. A statistically significant difference was observed in both groups after the intervention, indicating the positive effect of educational programs. Therefore, it seems that if the HBM is used in preventive behaviors of COVID-19 in pregnant women, it can lead to beneficial results for behavior change.

## Limitations

This study has some limitations. Firstly, since this study was conducted during the COVID-19 pandemic, the intervention was conducted through virtual networks, which might decrease the effect of educational programs. Secondly, a self-report questionnaire was used to collect data, which may have been subject to error and bias. The findings should be explained with caution when extrapolating to other countries. Studies are needed to be conducted among pregnant women in other countries in the future.

## Supplementary Information

**Additional file 1.** The questionnire used in the study to collect the data.

**Additional file 2.** Flow chart.

## Data Availability

The datasets used and/or analyzed during the current study are available from the corresponding author on reasonable request.

## Abbreviations

HBM: Health Belief Model
COVID-19: Coronavirus disease 2019
