# Supplementary material for: Investigating the effect of multimedia education based on the health belief model in preventing COVID-19 in pregnant women
Source: BMC Public Health. 2023 Apr 12;23:681. doi: 10.1186/s12889-022-14965-1 (PMC10090754; doi:10.1186/s12889-022-14965-1)
Supplement: Supplementary file 1 — Additional file 1. The questionnire used in the study to collect the data. [file 12889_2022_14965_MOESM1_ESM.doc]

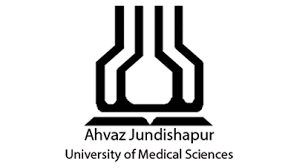


***Additional file 1***

Title of data: The questionnire used in the study to collect the data

**Date:** ......../......../..........

**Questionnaire code:** .................... **Name of the Health center:** ...............................

| **Dear, Respondent**  The present questionnaire is a part of research project entitled “The Efficiency of Health Belief Model Structures in Predicting the Preventive Behaviors of Pregnant Women over COVID-19 Pandemic. The results of the questionnaire will be beneficial for health care providers. Similarly, the results of the questionnaire will encourage the health promotion and disease prevention in pregnant women during Covid-19 pandemic. Please read each question carefully and answer as accurately as you can. It is noteworthy that the answers may be influenced by the respondents' experiences with the survey topic. There are no right or wrong answers, so please choose the survey responses that best describe your own experience. The questionnaire is anonymous and your responses will be kept strictly confidential.  **Ethics ID: IR.AJUMS.REC.1400.353** |
| --- |

**Part A.** Demographic information

**Q1. Age: ……………….…** year **Q2**. Gestational age **……………** weak

**Q3. Education Level:**

Under Diploma and Diploma 

Above Diploma and Masters 

Senior and Doctorate 

**Q4. Employment status**: Housewife  Worker  Employee Free  Others 

**Q5**- Have you injected the corona vaccine?

Yes  No

**Q6.** **If there was no injection, what was the reason?**

the fear From Complications🖵 Crowd of injection site🖵 No access🖵

No Knowledge🖵 Opposition from others and family🖵

**Part B. Please respond to the following questions by placing a check mark (√) in the answer box**

| number | Please read each question carefully and answer as accurately as you can. | YES | NO | I don'tknow |
| --- | --- | --- | --- | --- |
|  | **Category/Question** |  |  |  |
|  | **Knowledge** |  |  |  |
| 1 | The correct use of a mask effectively prevents the transmission of Coronavirus disease. |  |  |  |
| 2 | The mask must completely cover the nose and mouth. |  |  |  |
| 3 | As soon as the mask gets wet, it should be changed. |  |  |  |
| 4 | Do not lower the mask while talking. |  |  |  |
| 5 | The mask should not be touched and We can only touch the side strip when necessary |  |  |  |
| 6 | The minimum time for washing hands with soap and water is 20 seconds |  |  |  |
| 7 | The main way of transmission of Coronavirus disease is respiratory |  |  |  |
| 8 | Using proper nutrition is effective in preventing and controlling Coronavirus disease. |  |  |  |
| 9 | Pregnant women are high risk people for Coronavirus disease. |  |  |  |
| 10 | One of the most important ways to prevent Coronavirus is not to attend gatherings. |  |  |  |
| 11 | Proper ventilation plays an important role in preventing the transmission of Coronavirus disease. |  |  |  |
| 12 | The proper distance to observe the distance is 1/5 meters. |  |  |  |

**Part C. Please respond to the following questions by placing a check mark (√) in the answer box**

| *number* | *Please* read each question carefully and answer as accurately as you can. | **Strongly Agree** | **Agree** | **Neutral** | **Disagree** | **Strongly**  **Disagree** |
| --- | --- | --- | --- | --- | --- | --- |
|  | **Category/Question** |  |  |  |  |  |
|  | **Perceived Susceptibility** |  |  |  |  |  |
| 1 | There is a possibility that I will also get infected with Coronavirus disease during pregnancy. |  |  |  |  |  |
| 2 | Because I am a healthy pregnant woman, I guess I don'tneed to follow the health recommendations  Coronavirus disease. |  |  |  |  |  |
| 3 | Because I am pregnant, I follow the health more recommendations Coronavirus disease. |  |  |  |  |  |
| 4 | Being infected with Coronavirus disease during pregnancy makes the complications caused by this disease more severe |  |  |  |  |  |
| 5 | I am worried about complications and diseases caused by Coronavirus disease for my fetus. |  |  |  |  |  |
| **Perceived Severity** | | | | | | |
| 1 | If I get infected with Coronavirus disease, I may have more severe symptoms. |  |  |  |  |  |
| 2 | unknown complications of Coronavirus disease endanger the health of my fetus. |  |  |  |  |  |
| 3 | The pain and suffering of Coronavirus disease will be exhausting for me. |  |  |  |  |  |
| 4 | Because of my Coronavirus disease, I may even die. |  |  |  |  |  |
| 5 | The complications of Coronavirus disease can cause mental damage in me who is pregnant . |  |  |  |  |  |

**Part D. Please respond to the following questions by placing a check mark (√) in the answer box**

| number | Please read each question carefully and answer as accurately as you can. | **Strongly Agree** | **Agree** | **Neutral** | **Disagree** | **Strongly**  **Disagree** |
| --- | --- | --- | --- | --- | --- | --- |
|  | **Category/Question** |  |  |  |  |  |
|  | **Perceived Barriers** |  |  |  |  |  |
| 1 | I think that following the health recommendations against Corona is costly. |  |  |  |  |  |
| 2 | I think it will be time consuming for me to follow the health recommendations against Corona. |  |  |  |  |  |
| 3 | Frequent hand washing with soap and water and disinfecting surfaces makes my hands dry. |  |  |  |  |  |
| 4 | Using a mask will be boring for a pregnant woman. |  |  |  |  |  |
| 5 | Corona vaccine injection during pregnancy can be dangerous. |  |  |  |  |  |
| **Perceived benefits** | | | | | | |
| 1 | If I follow the health recommendations, the probability of me contracting Coronavirus disease is very small. |  |  |  |  |  |
| 2 | The cost of preventing Coronavirus disease is far less than the cost of treating it. |  |  |  |  |  |
| 3 | If I follow the health advice about Coronavirus disease, I will have a healthier pregnancy had. |  |  |  |  |  |
| 4 | Observing preventive behaviors Coronavirus disease will improve my physical health and that of my fetus |  |  |  |  |  |
| 5 | Following the health recommendations regarding Coronavirus disease can help improve the mental health of me and my family |  |  |  |  |  |

**Part E. Please respond to the following questions by placing a check mark (√) in the answer box**

| number | Please read each question carefully and answer as accurately as you can. | **Strongly Agree** | **Agree** | **Neutral** | **Disagree** | **Strongly**  **Disagree** |
| --- | --- | --- | --- | --- | --- | --- |
|  | **Category/Question** |  |  |  |  |  |
|  | **Self-efficacy** |  |  |  |  |  |
| 1 | Although the cost of procuring means to prevent coronavirus disease is high, but I can provide it. |  |  |  |  |  |
| 2 | I always cover my mouth and nose completely with a mask, even if it's boring. |  |  |  |  |  |
| 3 | Although washing my hands is time-consuming, I can do it. |  |  |  |  |  |
| 4 | Although disinfecting surfaces and equipment is time consuming and tedious, I am confident that I can do it well. |  |  |  |  |  |
| 5 | Although disinfecting and washing my hands regularly makes my skin dry, I can do it. |  |  |  |  |  |
| 6 | Although it is difficult to maintain distance from others outside the house, but I can maintain this Distance. |  |  |  |  |  |
| 7 | Because I am pregnant, I can refrain from attending parties and events for my health and that of my fetus. |  |  |  |  |  |

**Part F. Please respond to the following questions by placing a check mark (√) in the answer box**

| number | Please read each question carefully and answer as accurately as you can. | **Strongly Agree** | **Agree** | **Neutral** | **Disagree** | **Strongly**  **Disagree** |
| --- | --- | --- | --- | --- | --- | --- |
|  | **Category/Question** |  |  |  |  |  |
|  | **cues to action** |  |  |  |  |  |
| 1 | To get information on ways to prevent coronavirus disease, I take the advice of doctors and Health experts |  |  |  |  |  |
| 2 | I get help from social networks such as WhatsApp, Instagram and mobile phone SMS to get information on ways to prevent coronavirus disease. |  |  |  |  |  |
| 3 | I use radio and television to get information about ways to prevent corona disease |  |  |  |  |  |
| 4 | The fear of contracting coronavirus disease is the reason for me to follow the health recommendations. |  |  |  |  |  |
| 5 | Fear of harm to myself and my fetus is the reason for me to follow the health recommendations. |  |  |  |  |  |
| 6 | The fear of quarantine is the reason why I follow the health recommendations |  |  |  |  |  |

**part G. Please respond to the following questions by placing a check mark (√) in the answer box**

| number | Please read each question carefully and answer as accurately as you can. | **Always** | **Often** | **Sometimes** | **Rarely** | **Never** |
| --- | --- | --- | --- | --- | --- | --- |
|  | **Category/Question** |  |  |  |  |  |
|  | **Behavior questionnaire** |  |  |  |  |  |
| 1 | I will Use a disposable mask when leaving home |  |  |  |  |  |
| 2 | If my mask gets wet, I change it immediately |  |  |  |  |  |
| 3 | I wear a mask in a way that completely covers my nose and mouth. |  |  |  |  |  |
| 4 | I do not lower my mask when speaking. |  |  |  |  |  |
| 5 | I don't use torn, wide hole masks. |  |  |  |  |  |
| 6 | When applying and removing the mask, I only touch the side elastics. |  |  |  |  |  |
| 7 | We regularly wash our hands with soap and water for 15 to 20 seconds. |  |  |  |  |  |
| 8 | When washing hands, we carefully wash the lines of the palms and thumbs |  |  |  |  |  |
| 9 | When washing hands, we carefully wash the tips and between the fingers. |  |  |  |  |  |
| 10 | To greet and communicate with my friends and family, I use social networks such as WhatsApp and... |  |  |  |  |  |
| 11 | When leaving the house, I observe social distancing (distance of 1.5 meters from others on each side) |  |  |  |  |  |
| 12 | Because I am pregnant, I do not participate in family celebrations and ceremonies for the sake of my health and that of my fetus |  |  |  |  |  |
| 13 | Because I am pregnant, I try to shop online and offline. |  |  |  |  |  |
| 14 | I use telephone and internet consultations to receive prenatal care. |  |  |  |  |  |
| 15 | I avoid shaking hands and kissing when greeting others. |  |  |  |  |  |

We are extremely grateful for your contributing your valuable time, honest information, and thoughtful suggestions in identifying pregnant women health education needs. If you wish, write down your email or phone number so that we will share these results with you after completion of the research.

**Tel:** **Email:**
