# Supplementary material for: Investigating the effect of multimedia education based on the health belief model in preventing COVID-19 in pregnant women
Source: BMC Public Health. 2023 Apr 12;23:681. doi: 10.1186/s12889-022-14965-1 (PMC10090754; doi:10.1186/s12889-022-14965-1)
Supplement: Supplementary file 2 — Additional file 2. Flow chart. [file 12889_2022_14965_MOESM2_ESM.doc]

Flow chart

Selecting the control and intervention groups using a simple random sampling method and samples using cluster random sampling method

Referring to the health centers and preparing a list of pregnant women based on the inclusion and exclusion criteria from the existing electronic records

A phone call with pregnant mothers and send a link to complete the informed consent form

Sending the pre-test link to both intervention and control groups

Pre-test analysis

Content production based on the pre-test

تولید محتوا بر اساس پیش آزمون

تولید محتوا بر اساس پیش آزمون

Educational intervention in the intervention group

تولید محتوا بر اساس پیش آزمون

No educational intervention in the control group

مداخله آموزشی در گروه آزمون

تولید محتوا بر اساس پیش آزمون

مداخله آموزشی در گروه آزمون

Implementation of educational intervention

مداخله آموزشی در گروه آزمون

Sending a post-test link for both groups
